# Bicarbonate Inhibits Bacterial Growth and Biofilm Formation of Prevalent Cystic Fibrosis Pathogens

**DOI:** 10.3389/fmicb.2018.02245

**Published:** 2018-09-19

**Authors:** Orsolya Dobay, Krisztina Laub, Balázs Stercz, Adrienn Kéri, Bernadett Balázs, Adrienn Tóthpál, Szilvia Kardos, Pongsiri Jaikumpun, Kasidid Ruksakiet, Paul M. Quinton, Ákos Zsembery

**Affiliations:** ^1^Institute of Medical Microbiology, Semmelweis University, Budapest, Hungary; ^2^Department of Oral Biology, Semmelweis University, Budapest, Hungary; ^3^Department of Physiology, Semmelweis University, Budapest, Hungary; ^4^Department of Pediatrics, UC San Diego School of Medicine, University of California, San Diego, San Diego, CA, United States

**Keywords:** cystic fibrosis, bicarbonate, pH, biofilm, *Pseudomonas aeruginosa*, *Staphylococcus aureus*, cAMP, HCO_3_^−^

## Abstract

We investigated the effects of bicarbonate on the growth of several different bacteria as well as its effects on biofilm formation and intracellular cAMP concentration in *Pseudomonas aeruginosa*. Biofilm formation was examined in 96-well plates, with or without bicarbonate. The cAMP production of bacteria was measured by a commercial assay kit. We found that NaHCO_3_ (100 mmol l^-1^) significantly inhibited, whereas NaCl (100 mmol l^-1^) did not influence the growth of planktonic bacteria. MIC and MBC measurements indicated that the effect of ${\mathrm{HCO}}_{3}^{-}$ is bacteriostatic rather than bactericidal. Moreover, NaHCO_3_ prevented biofilm formation as a function of concentration. Bicarbonate and alkalinization of external pH induced a significant increase in intracellular cAMP levels. In conclusion, ${\mathrm{HCO}}_{3}^{-}$ impedes the planktonic growth of different bacteria and impedes biofilm formation by *P. aeruginosa* that is associated with increased intracellular cAMP production. These findings suggest that aerosol inhalation therapy with ${\mathrm{HCO}}_{3}^{-}$ solutions may help improve respiratory hygiene in patients with cystic fibrosis and possibly other chronically infected lung diseases.

## Introduction

Cystic fibrosis (CF) is caused by mutations in the gene encoding the cystic fibrosis transmembrane conductance regulator (CFTR) protein (Riordan et al., 1989). CFTR is a cAMP/protein kinase A (PKA)-dependent epithelial anion channel that conducts both chloride and bicarbonate (Linsdell et al., 1997; Reddy and Quinton, 2003). Defective transepithelial anion transport impairs mucociliary clearance (MCC) leading to the retention of thick, viscid mucus in the airways (Quinton, 2007a, 2010). The poor clearance of viscous CF mucus contributes to a vicious cycle of airway obstruction, infection, and inflammation (Hoffman and Ramsey, 2013). However, the links between the primary defect in anion transport and CF lung disease appear to be multifactorial. It was recently shown that impaired ${\mathrm{HCO}}_{3}^{-}$ secretion is likely responsible for aggregated mucus in CF mice (Garcia et al., 2009; Gustafsson et al., 2012) and pigs (Birket et al., 2014).

In addition, the abnormally lower pH of the airway surface liquid (ASL) was associated with decreased bacterial killing in the CF porcine lung. Aerosolizing a ${\mathrm{HCO}}_{3}^{-}$ solution onto the CF porcine airways increased innate bacterial killing *in vivo* (Pezzulo et al., 2012). Recruitment of lymphocytes may promote epithelial ${\mathrm{HCO}}_{3}^{-}$ secretion during infection and, intriguingly, epithelial ${\mathrm{HCO}}_{3}^{-}$ secretion may require CFTR expression in lymphocytes as yet another deficiency impacting on CF lung disease (Tang et al., 2012).

For decades, ${\mathrm{HCO}}_{3}^{-}$ has been used and indicated for use as a microbial disinfectant in food and agriculture industries, but usually as an adjuvant (Rutala et al., 2000). Earlier, ${\mathrm{HCO}}_{3}^{-}$, in combination with lidocaine was reported to exert antibacterial activity (Thompson et al., 1993). Immunologically, it is crucial for the optimal activity of antimicrobial peptides (Dorschner et al., 2006) but, apparently, it can have an independent bactericidal effect on *Escherichia coli* (Xie et al., 2010). In medicine, it is an accepted treatment for chlorine gas intoxication (Bosse, 1994) and has been suggested for use as a mucolytic as well as an adjuvant for nebulized drug delivery (Kaushik et al., 2016). It has also been long recommended for dental hygiene (Newbrun et al., 1984; Drake et al., 1995). Most of the antibacterial reports on ${\mathrm{HCO}}_{3}^{-}$ have been limited to planktonic growth and do not include effects on biofilm formation.

Bicarbonate directly and indirectly affects lung function (Quinton, 2007a,b). It is required for sequestering calcium and protons from secreted mucin granules to allow for normal expansion upon release (Garcia et al., 2009; Quinton, 2010). It may also affect neutrophil killing capacity as well as bacterial colonization in the lungs (Quinton, 2007a, 2008; Abou Alaiwa et al., 2014). Importantly, ${\mathrm{HCO}}_{3}^{-}$ influences the H^+^ concentration (pH) of the ASL via the ${\mathrm{HCO}}_{3}^{-}$/CO_2_ buffer system (Shah et al., 2016a). These effects directly affect the properties of the ASL and its critical ability to trap inhaled and endogenous debris for export by the ciliated surfaces of the airways. They indirectly affect the lungs by maintaining an airway environment that also enables the immune system to clear viral and bacterial pathogens. Any maneuver such as adding exogenous ${\mathrm{HCO}}_{3}^{-}$, as suggested here, that improves or helps maintain airway patency is expected to enhance lung function.

Successful antimicrobial therapy for bacterial lung infections is crucial for increasing life expectancy and improving CF patients’ quality of life. In CF lungs, however, bacteria colonize in biofilms (Rogers et al., 2011), making eradication of pathogens difficult. It is also known that bacteria in biofilms are more resistant compared to planktonic cells (Stewart and Costerton, 2001; Venkatesan et al., 2015). Biofilm formation is thought to be regulated largely by the second messenger molecules cAMP and c-di-GMP in bacterial cells. An increase in intracellular cAMP concentrations along with a decrease in c-di-GMP levels is associated with the production of acute virulence factors and reduced biofilm formation (Almblad et al., 2015). Accordingly, ${\mathrm{HCO}}_{3}^{-}$ stimulates soluble adenylate cyclase (sAC) and increases the activity of phosphodiesterase that breaks down c-di-GMP (Chen et al., 2000; Koestler and Waters, 2014a). Of note is that ${\mathrm{HCO}}_{3}^{-}$, CO_2_, and external pH affect soluble adenylate cyclase activity and consequently intracellular cAMP levels (Hammer et al., 2006), which is a hallmark for *Pseudomonas aeruginosa* virulence factors that decrease when bacteria form biofilms in chronic infection (Valentini and Filloux, 2016). Thus, bacterial life style (planktonic vs. biofilm) is, at least partially, dictated by intracellular cAMP levels.

Herein, we focus on the effects of ${\mathrm{HCO}}_{3}^{-}$ on planktonic growth on several pathogens common to cystic fibrosis and consider biofilm forming capacity for two of the more prevalent bacteria in CF, *P. aeruginosa* and *S. aureus*. This work presents a novel proposal that ${\mathrm{HCO}}_{3}^{-}$ may therapeutically help prevent colonization and biofilm formation of CF-associated bacteria by increasing intracellular cAMP levels. Not only do the data confirm earlier notions, but they also add further reasons to consider inhaled ${\mathrm{HCO}}_{3}^{-}$ as a potential therapy, especially in the context of CF where defective ${\mathrm{HCO}}_{3}^{-}$ secretion is a well-established basic defect.

## Materials and Methods

### Growth Experiments

The growth of ATCC control strains and clinical isolates of different Gram-positive and Gram-negative bacteria in Brain-Heart Infusion (BHI) broth (Mast Group Ltd., Merseyside, United Kingdom) were compared with and without 100 mmol l^-1^ NaHCO_3_ in the medium and equilibrated with CO_2_ to control pH. Growth experiments were carried out with the following reference strains: *S. aureus* ATCC 25923 (MSSA), *S. aureus* ATCC 29213 (MSSA), *S. aureus* ATCC 33591 (MRSA), *Streptococcus agalactiae* ATCC 80200, *Enterococcus faecalis* ATCC 29212, *E. faecalis*, vanB+, ATCC 51299, *P. aeruginosa* ATCC 27853, *E. coli* ATCC 25922, *Haemophilus influenzae* ATCC 49766, *H. influenzae* ATCC 49247. Some of the experiments were repeated with clinical isolates of the same species, obtained from the Central Bacteriological Diagnostic Laboratory of Semmelweis University, Budapest, obtained from daily routine specimens.

The density of bacterial suspensions was set at 0.5 McFarland (approximately 10^8^ CFU ml^-1^) with a VITEK Densichek apparatus (Biomérieux, Marcy l’Etoile, France). The pH of the 100 mmol l^-1^ NaHCO_3_-containing BHI broth was set at pH approximately 7.4 by bubbling the autoclaved solution to equilibration with 20% CO_2_-balance air for at least 16 h at 37°C before inoculation. The pH of these unstirred solutions in hermetically capped bottles remained stable for at least 24 h. To achieve other pH values, the CO_2_ concentration required was calculated from the Henderson–Hasselbalch equation for the ${\mathrm{HCO}}_{3}^{-}$/CO_2_ buffer system (Story, 2004). When BHI broth containing 100 mmol l^-1^ NaHCO_3_ was equilibrated with 5% CO_2_, the measured pH (∼8.5) was somewhat higher than that calculated from the Henderson–Hasselbalch equation (pH ∼8.0).

Aliquots of each suspension (200 μl) were dispensed into 96-well microtiter plates in duplicate. Bacterial suspensions were then incubated in ambient air (∼0.04% CO_2_), in 5% or in 20% CO_2_ by design. Bacterial growth was followed by measuring the optical density (OD) at 595 nm using a PR2100 microplate reader (Bio-Rad Laboratories, Hercules, Canada) 60 min after inoculating and subsequently every 15 min for 5.5 h. Optical density of a negative control (without bacterial growth) was subtracted from all OD values. The results of the parallel measurements of duplicate samples were averaged and normalized to the control media. The growth rates were determined by calculating the area under the curve (AUC) (Horváth et al., 2012) using Microsoft Excel, based on the summation of small trapezoids.

The osmolality of BHI broth was approximately 360 mosm kg^-1^ in accordance with the data published earlier (Montgomerie et al., 1972). Since supplementation of BHI broth with 100 mmol l^-1^ NaHCO_3_ increased the osmolality of the solution to approximately 470 mosm kg^-1^, bacterial growth was also determined in BHI broth containing 100 mmol l^-1^ NaCl (pH 7.4 adjusted with NaOH) as a control. As a pH control, the growth of bacteria was measured in unsupplemented BHI broths adjusted with HCl or NaOH to pH values ranging from 6.8 to 9.0 as required.

### Determination of Minimum Inhibitory Concentration (MIC) and Minimum Bactericidal Concentration (MBC) by Broth Microdilution

To determine MIC and MBC, two strains of *P. aeruginosa* (ATCC 27853 and a clinical isolate) and two strains of *S. aureus* (ATCC 29213 and a clinical isolate) were selected. Bacteria were cultured for 18-20 hours on blood agar plates. The following day, a 0.5 McFarland suspension was prepared in physiological salt solution. This suspension was diluted 1:20 in Mueller–Hinton (MH) Broth (cation adjusted). For further measurements this standardized inoculum was used. NaHCO_3_ was diluted serially twofold in MH Broth (cation adjusted) in a plastic microdilution plate with round bottom wells to a final volume of 0.1 ml. The concentration of NaHCO_3_ varied from 1000 to 1.95 mmol l^-1^ in 10 steps of twofold dilutions. The wells were inoculated with 0.01 ml of standardized inoculum, positive and negative growth controls were also used. After 24 h incubation, the growth of bacteria was evaluated based on the visible change of turbidity. MIC was identified as the lowest concentration of HCO_3_ at which no visible growth was observed. To determine MBC, specimens from the wells without visible bacterial growth were inoculated onto antibiotic-free agar plates and incubated for 24 h. MBC was defined as the lowest concentration of HCO_3_ where no colonies were observed.

### Biofilm Experiments

Although almost all bacteria involved in CF lung disease can form biofilms, *P. aeruginosa* presents the largest clinical challenge. Therefore, we investigated *P. aeruginosa* for biofilm formation. Isolates were grown overnight to stationary phase in bouillon containing meat extract 0.3%, yeast extract 0.2%, pepton 1%, NaCl 0.5%; pH adjusted to 7.5 with NaOH. The overnight cultures were diluted 1:100 in the desired medium. All solutions were prepared by filtration using 0.22 μm filter membranes. Aliquots of 100 μl of diluted cultures were pipetted into eight parallel wells of a 96-well microtiter plate. The covered plates were incubated at 37°C in ambient air, 5%, or 20% CO_2_ for 48 h. Planktonic bacteria were then removed by rigorous washing with PBS three times. Bacteria attached to the wells were air-dried and subsequently stained with 125 μl of 0.1% crystal violet solution for 10 min. Excess crystal violet was removed by water-washing; that is, submerging the plates in tap water several times. After air-drying, crystal violet was solubilized in 30% acetic acid (200 μl per well) for 10 min. From each well, 125 μl of this solution was then transferred to separate wells of an optically clear, flat-bottom 96-well plate. Optical density was measured at 595 nm in a PR2100 microplate reader (see above) (Merritt et al., 2011).

To measure the influence of glucose-content on biofilm formation, the bouillon was supplemented with 0.2, 1.0, 2.0, and 4.0 g l^-1^ glucose. Biofilm formation was absent at low glucose content and was the strongest at 4.0 g l^-1^ glucose, consequently all experiments were performed using bouillon containing 4.0 g l^-1^ glucose.

### Measurement of Intracellular cAMP Levels

The bacterial production of cAMP was determined with the Cyclic AMP XP^®^ Assay Kit (Cell Signaling Technology, Leiden, Netherlands, originally designed for eukaryotic cells, but applicable for bacteria as well). Bacterial cultures were first grown in the desired medium for 15–16 h to stationary phase for conditioning, then diluted 1:500 in 10 ml of the same fresh medium and allowed to grow for a few hours until reaching log phase, that is, OD_595_ = 0.4–0.5. Cultures were then centrifuged (4000 × *g*, 5 min), bacteria were re-suspended in 200 μl of the kit lysis buffer for 30 min on ice and centrifuged again (8000 × *g*, 5 min); 50 μl of the supernatant was transferred to the assay plate. According to kit protocol, absorbance was measured at 450 nm in a PR2100 microplate reader.

### Statistical Analysis

For statistical analysis Statistica for Windows 7.0 (Statsoft) was used. Data presented are means ± SD, if not indicated otherwise. The values were compared using ANOVA followed by LSD *post hoc* comparison test. Changes were considered statistically significant at *P* < 0.05.

## Results

### Reduction of Bacterial Growth by Bicarbonate

**FIGURE 1:**
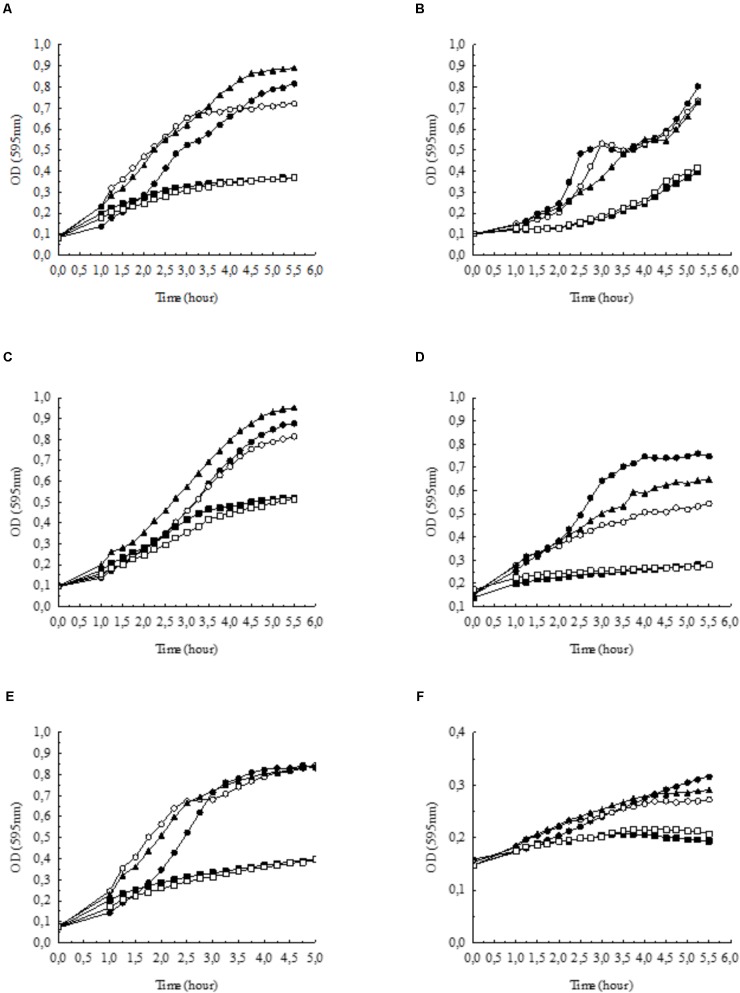
Growth of **(A)** *Escherichia coli*, **(B)** *Pseudomonas aeruginosa*, **(C)** *Staphylococcus aureus*, **(D)** *Streptococcus agalactiae*, **(E)** *Enterococcus faecalis*, and **(F)** *Haemophilus influenzae* in BHI broth supplemented with NaHCO_3_ compared to control conditions (•: control pH = 7.4, 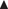: 100 mmol l^-1^ NaCl, pH = 7.4). Bicarbonate-containing solutions were equilibrated with either 5% (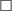: 100 mmol l^-1^ NaHCO_3_, pH = 8.5) or 20% CO_2_ (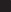: 100 mmol l^-1^ NaHCO_3_, pH = 7.4). In the absence of bicarbonate, pH was adjusted to 8.5 with NaOH (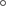: growth at pH = 8.5). Each curve shows the average of two parallel experiments. Standard Deviations were generally less than 1% of the mean and are not shown. Note that in panel **(F)** the scale on the *y* axis differs from the other panels.

Since high external ${\mathrm{HCO}}_{3}^{-}$ concentrations and/or alkaline pH are reported to inhibit the growth of *E. coli* (Xie et al., 2010), we tested the effect of ${\mathrm{HCO}}_{3}^{-}$ on the growth of *E. coli*. The growth rate of bacteria was significantly inhibited in BHI broth supplemented with 100 mmol l^-1^ NaHCO_3_ (pH 7.4, equilibrated with 20% CO_2_) as compared to control media (pH 7.4) without added ${\mathrm{HCO}}_{3}^{-}$ (**Figure 1A**). We tested the growth of bacteria in BHI broth supplemented with 100 mmol l^-1^ NaCl (pH 7.4; ∼ Δ110 mosm kg^-1^), which did not suppress the growth of bacteria, indicating that the inhibitory effect of NaHCO_3_ was not due to increased osmolality or ionic strength.

Next, we tested whether alkaline pH affects bacterial growth. In BHI broth at pH 8.5, the growth capacity was not significantly influenced (**Figure 1A**); however, when bacteria were incubated in BHI broth supplemented with 100 mmol l^-1^ NaHCO_3_ at pH 8.5 (5% CO_2_), the inhibitory effects were similar to those observed at pH 7.4 (20% CO_2_) (**Figure 1A**). Thus, the data show that NaHCO_3_ *per se* inhibited bacterial growth, which was not simply due to higher osmolality or alkaline media.

In order to test whether the inhibitory effect of HCO_3_ is specific to *E. coli* cells, we investigated other species such as *P. aeruginosa, S. aureus, S. agalactiae, E. faecalis*, and *H. influenzae*. Similarly to the effects on *E. coli*, NaHCO_3_ (100 mmol l^-1^) significantly inhibited the growth of all these species as well, suggesting that ${\mathrm{HCO}}_{3}^{-}$ can suppress bacterial growth in general (**Figures 1B–F**). In order to compare the growth rates of bacteria under different conditions more quantitatively, AUC values were determined as a measure of the reduced growth rates in ${\mathrm{HCO}}_{3}^{-}$-enriched medium for each bacterial specie (**Table 1**).

**Table 1:** Calculated AUC values of different bacteria, based on growth curves in **Figures 1A–F**.

Bacterium	Growth control pH = 7.4	Growth at pH = 8.5	100 mM NaCl pH = 7.4	100 mM NaHCO_3_ pH = 7.4	100 mM NaHCO_3_ pH = 8.5
*E. coli*	2.45	2.82	3.04	1.57	1.49
*P. aeruginosa*	2.05	1.92	1.82	1.01	1.06
*S. aureus*	2.48	2.41	2.93	1.92	1.76
*S. agalactiae*	2.84	2.20	2.48	1.27	1.35
*E. faecalis*	2.90	3.21	3.18	1.64	1.54
*H. influenzae*	1.28	1.26	1.31	1.05	1.07


**FIGURE 2:**
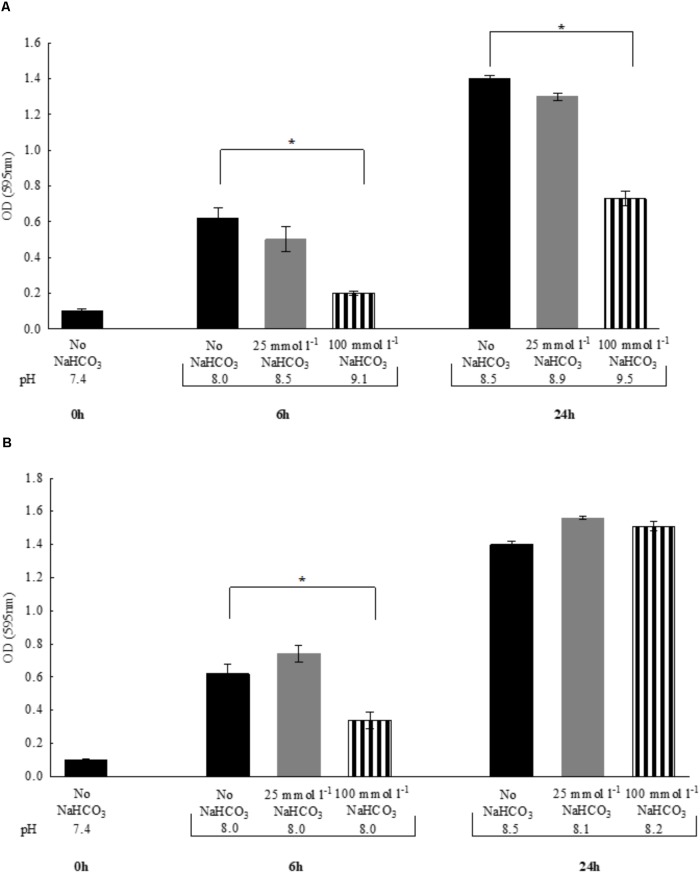
Changes in bacterial density of *Pseudomonas aeruginosa* and media pH following 6 vs. 24 h incubations either **(A)** in air atmosphere, or **(B)** in the presence of appropriate levels of CO_2_. Black bars: NaHCO_3_-free controls, gray bars: 25 mmol l^-1^ NaHCO_3_; striped bars: 100 mmol l^-1^ NaHCO_3_. Statistically significant, ^∗^*P* < 0.05.

**FIGURE 3:**
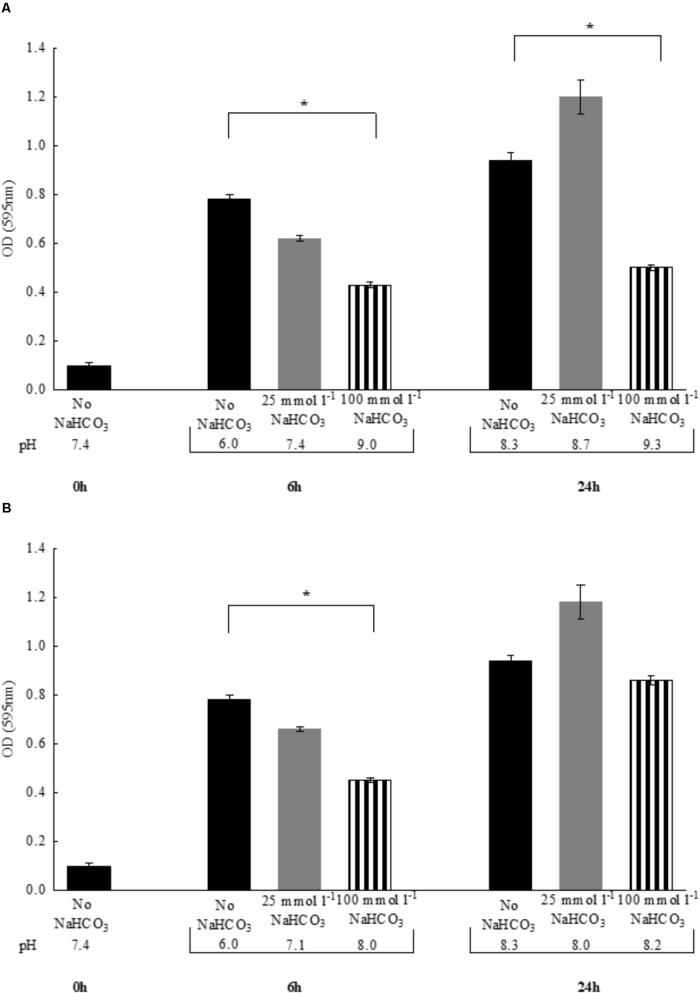
Changes in bacterial density of *Staphylococcus aureus* and media pH following 6 vs. 24 h incubations either **(A)** in air atmosphere or **(B)** in the presence of appropriate CO_2_ levels. Black bars: NaHCO_3_-free controls, gray bars: 25 mmol l^-1^ NaHCO_3_, striped bars: 100 mmol l^-1^ NaHCO_3_. ^∗^*P* < 0.05.

In the previous experiments, to maintain the initial pH of the media near 7.4, all bicarbonate-containing solutions were equilibrated with calculated levels of CO_2_. Therefore, we asked the question whether ${\mathrm{HCO}}_{3}^{-}$-containing media without CO_2_ equilibration influences growth of *P. aeruginosa*. Despite the fact that media pH continued to increase during the exponential growth phase (i.e., the first six hours), bacterial density remained similar to those observed with CO_2_ equilibration (**Figures 2A,B**). When bacteria were grown until reaching the stationary phase (i.e., 24 h), significant increases in OD values were observed in the presence of both 25 and 100 mmol l^-1^ ${\mathrm{HCO}}_{3}^{-}$ suggesting that ${\mathrm{HCO}}_{3}^{-}$ is bacteriostatic rather than bactericidal. Of note is that a lower bacterial density was observed at 24 h when 100 mmol l^-1^ ${\mathrm{HCO}}_{3}^{-}$ was present, but it should be kept in mind that excessive alkalinization at atmospheric CO_2_ levels, may exert inhibitory effects on growth rate (**Figure 2A**). Nonetheless, ${\mathrm{HCO}}_{3}^{-}$-containing media of concentrations up to 100 mmol l^-1^ equilibrated with appropriate levels of CO_2_ did not inhibit maximal bacterial growth at 24 h, suggesting that the bacteriostatic effects of ${\mathrm{HCO}}_{3}^{-}$ occur within the initial period of the exponential growth phase (**Figure 2B**). We obtained similar results when the above experiments were repeated with *S. aureus*, at least with respect to bacterial growth profile (**Figures 3A,B**).

### Results of the MIC and MBC Determination

In the case of all four tested isolates, an MIC of 125 mmol l^-1^ was obtained. The MBCs for the two *P. aeruginosa* strains were 500 mmol l^-1^, meanwhile the two *S. aureus* isolates remained alive even in the highest ${\mathrm{HCO}}_{3}^{-}$ concentration tested (MBC > 1000 mmol l^-1^).

### Bicarbonate Inhibits Biofilm Formation of *P. aeruginosa*

**FIGURE 4:**
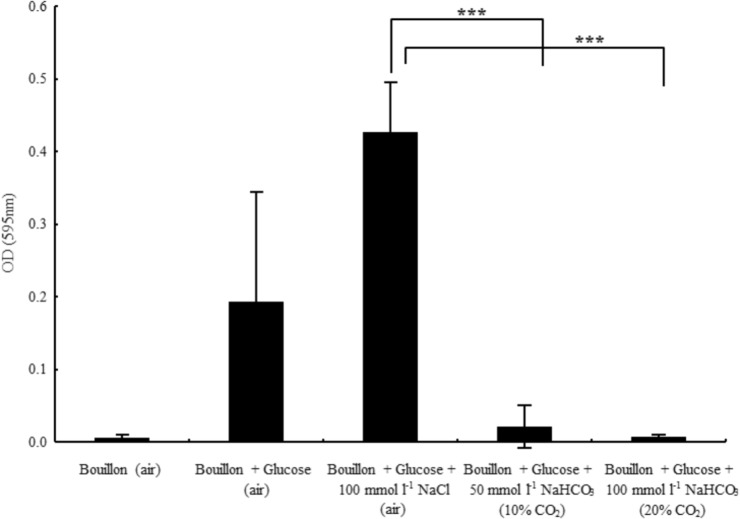
Biofilm formation capacity of *Pseudomonas aeruginosa* in glucose-containing bouillon (4 g l^-1^) in the presence of sodium chloride and two different concentrations of sodium bicarbonate. Please note that in the absence of glucose no biofilm formation was observed. ^∗∗∗^*P* < 0.001.

One of the most severe complications of CF lung disease involves biofilm formation of pathogenic bacteria, which may be due to depleted levels of ${\mathrm{HCO}}_{3}^{-}$ in CF airways. Thus, we asked the question whether ${\mathrm{HCO}}_{3}^{-}$ could not only inhibit planktonic bacterial growth, but biofilm formation as well. Glucose starvation leads to impaired biofilm formation associated with elevated cAMP levels (Huynh et al., 2012), hence glucose is required for optimal biofilm formation. Similarly, our data show that although bacteria achieved high density (OD_595_ = 0.94 ± 0.12, *n* = 7), no biofilm was detected in the medium without added glucose (**Figure 4**). On the other hand, in the presence of glucose, bacterial growth was similar (OD_595_ = 0.81 ± 0.06, *n* = 7) and robust biofilm formation was observed. However, the supplementation of glucose-containing medium with 100 mmol l^-1^ ${\mathrm{HCO}}_{3}^{-}$ prevented biofilm formation and significantly inhibited planktonic growth as well (OD_595_ = 0.45 ± 0.04, *n* = 8). To demonstrate the reduced number of viable cells in ${\mathrm{HCO}}_{3}^{-}$-containing media, a bacteria count was performed after 48 h’ incubation. In the absence of ${\mathrm{HCO}}_{3}^{-}$, the calculated CFU in bouillon with and without glucose was 2.8 × 10^12^ and 4.5 × 10^11^, respectively. In contrast, the average CFU ml^-1^ was 6.6 × 10^6^ in media supplemented with 100 mmol l^-1^ ${\mathrm{HCO}}_{3}^{-}$. In addition, 50 mmol l^-1^ ${\mathrm{HCO}}_{3}^{-}$ partially blocked biofilm formation suggesting a concentration-dependent inhibitory mechanism (**Figure 4**). When NaCl (100 mmol l^-1^) replaced NaHCO_3_, biofilm formation capacity was fully maintained.

### Bicarbonate Increases Intracellular cAMP Levels in Bacteria

**FIGURE 5:**
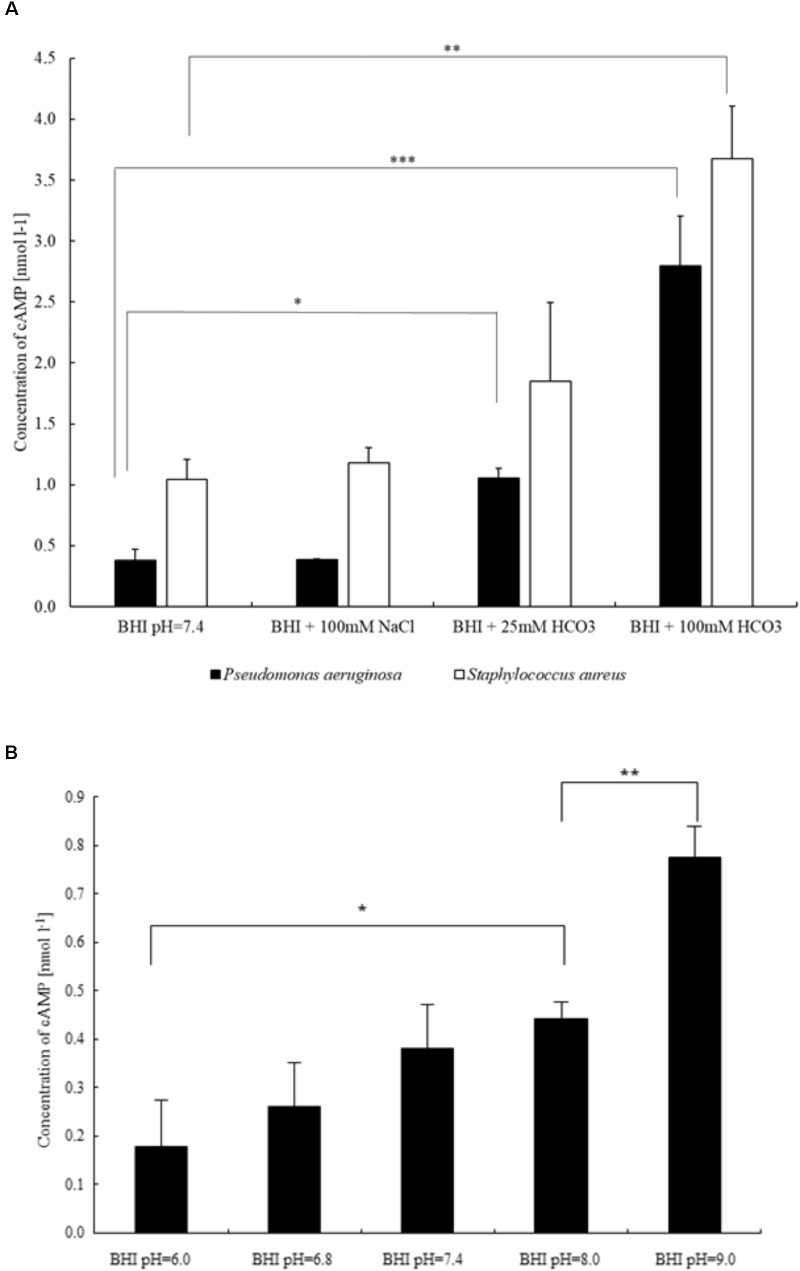
Differences in intracellular cAMP production of bacteria influenced by **(A)** sodium chloride and two different concentrations of sodium bicarbonate (*P. aeruginosa* and *S. aureus*), and **(B)** external pH (*P. aeruginosa*). ^∗^*P* < 0.05, ^∗∗^*P* < 0.01, ^∗∗∗^*P* < 0.001.

Inhibition of biofilm formation may be induced by ${\mathrm{HCO}}_{3}^{-}$ effects on the levels of cAMP and c-di-GMP. Thus, we investigated the effects of ${\mathrm{HCO}}_{3}^{-}$ on intracellular cAMP production in *P. aeruginosa*. Supplementation of BHI medium with 25 and 100 mmol l^-1^ ${\mathrm{HCO}}_{3}^{-}$ resulted in significant concentration-dependent increases in cAMP levels (**Figure 5A**). Importantly, administration of 100 mmol l^-1^ NaCl did not influence cAMP concentrations (**Figure 5A**). Since it is well known that sAC activity depends on intracellular pH (Rahman et al., 2013), we tested the effects of media pH changes between 6.0 and 9.0 on cAMP production. In this pH range, a slight increase in cAMP levels, parallel to alkalinization was observed (**Figure 5B**). Of note, even the highest pH-induced increase in cAMP level was significantly lower than the increase induced by 100 mmol l^-1^ ${\mathrm{HCO}}_{3}^{-}$. Similar results were obtained when the experiments were repeated with *S. aureus* (**Figure 5A**). Taken together, the data suggest that biofilm suppression by ${\mathrm{HCO}}_{3}^{-}$ is mediated through an increased production of intracellular cAMP.

## Discussion

Early studies using the human serous cell line (Calu-3) showed that airway-derived epithelial cells can secrete ${\mathrm{HCO}}_{3}^{-}$ (Lee et al., 1998). Recently, active ${\mathrm{HCO}}_{3}^{-}$ secretion was demonstrated in native intact small airway epithelium as well (Shamsuddin and Quinton, 2014). Loss of ${\mathrm{HCO}}_{3}^{-}$ secretion seems to be associated with different pathological consequences in CF airways, such as formation of thickened mucus (Quinton, 2010), reduced microbial susceptibility to antimicrobial peptides (Dorschner et al., 2006) and impaired bacterial killing capacity (Pezzulo et al., 2012; Abou Alaiwa et al., 2014). Therefore, the delivery of ${\mathrm{HCO}}_{3}^{-}$ into the airways may be potentially therapeutic in CF (Pezzulo et al., 2012; Li et al., 2016). Moreover, beyond CF, decreased pH (likely due to decreased ${\mathrm{HCO}}_{3}^{-}$) of airway surface liquid and chronic bacterial infections have also been described in other chronic airway diseases such as COPD (Mall and Hartl, 2014; Li et al., 2016; Shah et al., 2016a,b).

A shift to either acidic or alkaline external pH presents a stress for bacteria, which may influence survival and growth (Padan et al., 2005). In alkaline environments the growth rate of neutrophilic bacteria is reduced (Maurer et al., 2005). When grown in media at pH 7.0, *E. coli* cells exhibit shorter generation time compared to that when it is cultured at pH 8.7 (Maurer et al., 2005). Our data show that 100 mmol l^-1^ NaHCO_3_ significantly inhibit the growth of *E. coli* equally, at both pH 7.4 and 8.5, but in the absence of ${\mathrm{HCO}}_{3}^{-}$, neither alkaline pH (up to 8.5) nor equivalent increases in osmolality (NaCl) inhibit bacterial growth, indicating that ${\mathrm{HCO}}_{3}^{-}$ *per se* plays a pivotal role in growth suppression. Alkaline conditions may alter cytosolic pH of bacteria when protons must be taken up from the extracellular medium. Under alkaline conditions proton scavengers markedly decrease *E. coli* viability (Vanhauteghem et al., 2013). This phenomenon can be explained by partitioning of unprotonated scavengers into the cytosol where they become protonated and increase cytosolic pH. To maintain pH homeostasis, bacteria require high ATP consumption and membrane hyperpolarization. We surmise that higher ${\mathrm{HCO}}_{3}^{-}$ concentrations in the media increase the gradient for increased ${\mathrm{HCO}}_{3}^{-}$ entry into the cytosol of bacteria elevating intracellular pH. The higher energy consumption occurs at the expense of bacterial growth rate. Importantly, similar results were observed with *S. aureus, P. aeruginosa, S. agalactiae, E. faecalis*, and *H. influenzae* as well, again suggesting that ${\mathrm{HCO}}_{3}^{-}$ might be used to inhibit the growth of bacteria more generally.

*S. aureus* and *P. aeruginosa* are of particular interest because of their high incidence in CF patients with chronic pulmonary infections. We observed that during the first few hours of growth *S. aureus* decreased, whereas *P. aeruginosa* increased the pH of the medium. These data suggest that ${\mathrm{HCO}}_{3}^{-}$, rather than pH shifts, suppressed bacterial growth. In *P. aeruginosa* these observations were confirmed in ${\mathrm{HCO}}_{3}^{-}$-containing media without CO_2_ equilibration where despite the increase in the pH of the media, the growth rate was similar to that detected with CO_2_ equilibration. Extending the incubation to 24 h clearly demonstrated that the effects of ${\mathrm{HCO}}_{3}^{-}$ were bacteriostatic rather than bactericidal. Furthermore, in the absence of CO_2_ a critically high pH (>9.2) occurred with a reduced growth rate.

Recurrent lung infections caused by long-term colonization of biofilm-forming bacteria are a significant threat for CF patients (Høiby et al., 2017). Importantly, NaHCO_3_ disrupts oral biofilms *in vitro* (Pratten et al., 2016). NaHCO_3_ in combination with sodium metaperiodate and sodium dodecyl sulfate also suppressed the formation of *P. aeruginosa* biofilms (Gawande et al., 2008). We also confirmed these observations as NaHCO_3_ prevented biofilm formation at 100 mmol l^-1^ and inhibited the planktonic growth of bacteria. Since the administration of equimolar NaCl (100 mmol l^-1^) had no effect and lower concentrations of NaHCO_3_ only partially blocked biofilm formation, we concluded that in our experimental model ${\mathrm{HCO}}_{3}^{-}$ suppresses bacterial conversion to biofilms as a function of concentration. These observations may be therapeutically promising as our unpublished data suggest that 100 mmol l^-1^ NaHCO_3_ does not have toxic effects on airway epithelial cells *in vitro*.

Biofilm formation requires coordination between cAMP and c-di-GMP signaling in several bacteria. In *P. aeruginosa* increased levels of c-di-GMP suppress signaling from the cAMP-virulence factor regulator pathway and the expression of virulence factors that favor a persistent biofilm state (Almblad et al., 2015). On the other hand, cAMP stimulates the production of acute virulence factors, but inhibits biofilm formation in *Vibrio cholerae* (McDonough and Rodriguez, 2011). Likewise, ${\mathrm{HCO}}_{3}^{-}$ also activates virulence gene expression in *V. cholerae* (Iwanaga and Yamamoto, 1985; Abuaita and Withey, 2009). In the intestinal lumen, ${\mathrm{HCO}}_{3}^{-}$ apparently suppresses the bile-mediated induction of c-di-GMP that inhibits biofilm formation (Koestler and Waters, 2014b). Based on the above observations, we speculated that ${\mathrm{HCO}}_{3}^{-}$ should increase bacterial cAMP levels. In fact, our data show that NaHCO_3_ stimulates intracellular cAMP concentrations in both *P. aeruginosa* and *S. aureus*. The stimulatory effect was not observed in the presence of equivalent NaCl concentrations indicating a specific role for ${\mathrm{HCO}}_{3}^{-}$. Interestingly, however, cAMP production was dependent on external pH in the range between 6.0 and 9.0. These results are consistent with the findings that sAC is regulated by various environmental signals such as calcium and CO_2_/${\mathrm{HCO}}_{3}^{-}$/pH (McDonough and Rodriguez, 2011; Rahman et al., 2013). Chen et al. (2000) previously demonstrated that sAC functions as a ${\mathrm{HCO}}_{3}^{-}$ sensor in many biological systems. More recently, ${\mathrm{HCO}}_{3}^{-}$ has been shown to increase cAMP production via sAC stimulation in corals (Barott et al., 2013). Thus, we surmise that reduced ${\mathrm{HCO}}_{3}^{-}$ secretion from CF lung epithelial cells results in decreased luminal pH, which leads to decreased levels of cAMP production in bacterial cells. Low bacterial cAMP levels reduce virulence factor production, and thus, fail to alert the innate host immune systems, allowing for more favorable conditions for biofilm formation. Parallel to these events, elevated concentrations of c-di-GMP may also support biofilm formation, making eradication of bacteria from the airways difficult.

## Conclusion

Since we found that increased ${\mathrm{HCO}}_{3}^{-}$ impedes the growth and biofilm formation of several pulmonary bacterial pathogens, we expect that increasing ${\mathrm{HCO}}_{3}^{-}$ in the airways may reduce infection, inflammation, and a consequent tissue damage in the lungs. We assume that the inhalation of aerosolized ${\mathrm{HCO}}_{3}^{-}$ could prove therapeutic against bacteria such as *S. aureus* and *P. aeruginosa*, which are among the most relevant pathogens in lung infections in CF. Although inhalation therapy is inherently episodic and therefore the concentration of ${\mathrm{HCO}}_{3}^{-}$ on the airways is certain to dissipate between inhalation intervals, the therapy may offer significant benefits even with only acute changes in the airway surface liquid composition by depressing bacterial growth repetitively and by increasing the ability of the innate immune system to reduce or clear the infection load. However, caution should be taken with treatments that may affect intracellular cAMP levels because increasing cAMP may enhance the expression of virulence factors and lead to acute exacerbation in CF patients with chronic bacterial infections (Coggan and Wolfgang, 2012). Routinely, CF patients are treated repeatedly with different courses of antibiotics in spite of growing concerns over antibiotic side effects. Although antagonistic effects of ${\mathrm{HCO}}_{3}^{-}$ have been reported for tobramycin efficacy (Kaushik et al., 2016), our unpublished preliminary data suggest that the efficacy of both erythromycin and imipenem increases in an alkaline environment. Based on these observations, ${\mathrm{HCO}}_{3}^{-}$, inhaled regularly, may reduce use of antibiotics.

## Author Contributions

OD, KL, BS, AK, BB, AT, SK, PJ, KR, and ÁZ were involved in lab experiments. PQ, ÁZ, and DO designed the study. ÁZ, DO, KL, and BS analyzed the data. ÁZ wrote the manuscript. PQ reviewed and critically revised the manuscript.

## Conflict of Interest Statement

The authors declare that the research was conducted in the absence of any commercial or financial relationships that could be construed as a potential conflict of interest.
